# An unusual case of huge fibrotic sac of hematoma at saphenous vein harvest site for coronary artery bypass grafting: a case report

**DOI:** 10.1186/s13019-023-02324-z

**Published:** 2023-07-04

**Authors:** June Lee, Hye Rim Na, Seok Beom Hong, Do Yeon Kim, Hwan Wook Kim, Yong Han Kim

**Affiliations:** Department of Thoracic and Cardiovascular Surgery, Seoul St. Mary’s Hospital, College of Medicine, The Catholic University of Korea, 222 Banpo- daero, Seocho-gu, Seoul, 06591 Republic of Korea

**Keywords:** Hematoma, Coronary artery bypass grafting, Saphenous vein harvest

## Abstract

Great saphenous vein is a conduit commonly used for coronary artery bypass grafting. However, several complications could occur at leg wound site for vein harvesting. Here, we describe a huge sac of hematoma as an uncommon complication of saphenous vein harvest for coronary artery bypass grafting.

A 62-year-old gentleman was readmitted with swelling at left thigh 30 days after coronary artery bypass grafting. Lower extremity computed tomography was suggestive of an oval and thick sac implying a hematoma or seroma. After using ultrasound scanning for the mass, an incision through the previous surgical wound showed a huge mass. Inspection after incision the mass revealed an old hematoma within the sac.

Pathologic findings demonstrated chronic inflammation with the hematoma surrounded by a fibrotic sac. The patient’s postoperative course was uneventful without recurrence.

Our experience suggests the possibility of a huge hematoma within a thick fibrotic sac at the previous vein harvest site for coronary artery bypass grafting.

## Introduction

Great saphenous vein (GSV) is widely used as a conduit for coronary artery bypass grafting (CABG) [1]. However, 2.4–17.7% of patients show complications including infection at the vein harvest site [2, 3]. Diabetes mellitus and many other risk factors for surgical wound infection of saphenous vein harvest have been reported [2]. Besides surgical site infection, wound gap, bleeding, hematoma, edema, serous discharge, pain, and erythema are also surgical wound complications of saphenous vein harvest [4].

Attempts have been made to reduce leg wound complications with minimally invasive approach including endoscopic vein harvest [5, 6]. Preoperative ultrasound scanning for GSV mapping to predict anatomy of the vein can also be used to minimize leg wound incision and reduce harvest time with the benefit of decreasing wound complications rate [7]. There is still controversy regarding the frequency of complications at the incision site in the leg and the no-touch saphenous vein harvesting technique [8, 9].

Herein, we describe a huge sac of hematoma as an uncommon complication of saphenous vein harvest for CABG.

## Case presentation

**Fig. 1:**
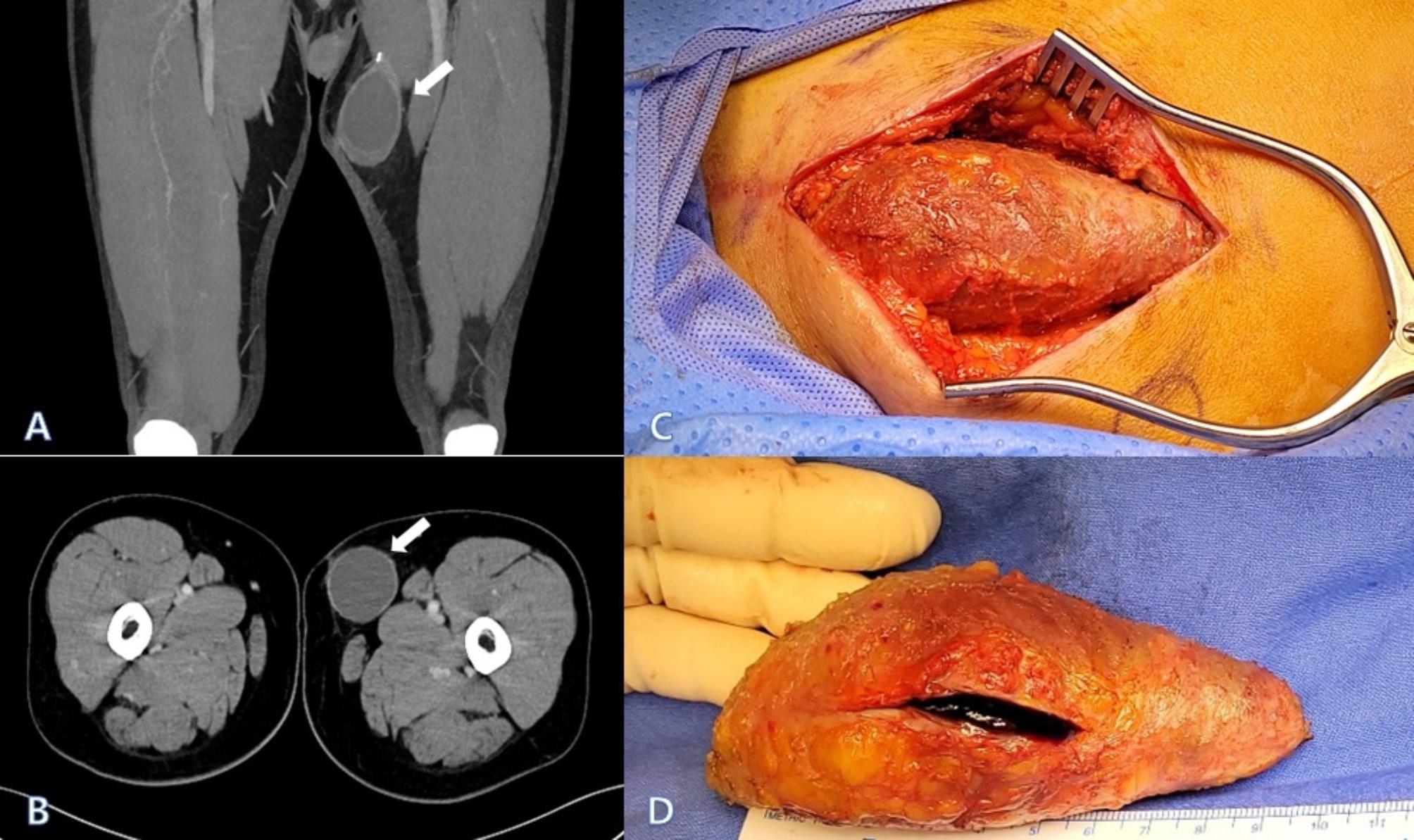
(**A**) Initial lower extremity CT revealing a capsulated mass implying hematoma or seroma (white arrow). (**B**) Axial view of the lower extremity CT showing the mass (white arrow). (**C**) Intraoperative findings showing a mass. (**D**) The thick fibrotic sac containing hematoma

A 62-year-old gentleman presented to the outpatient department with swelling of left thigh. He had a history of CABG surgery using open vein harvesting 30 days ago at our center and was a current smoker until he underwent the heart surgery. Through two longitudinal incisions at left thigh, the saphenous vein was extracted by ‘no touch’ technique. The saphenous vein pedicle, along with the saphenous fascia encompassing the graft and a margin of adjacent adipose tissue measuring more than 5 mm, was harvested. All branches of the vein were ligated using titanium clips (Teleflex Medical, 3015 Carrington Mill Boulevard, Morrisville, NC 27,560, USA). The leg wounds were closed layer by layer, without the use of a drainage tube. Postoperatively, a pressure dressing was applied to the surgical site. Aspirin and clopidogrel was given to the patient after the surgery. He was discharged without any problems including the leg wound site. However, he experienced a slip down injury while walking at two weeks after surgery. On examination, there was a large mass of the left thigh along the prior vein harvest site. The oval shaped mass was not soft. It was firm and fixed. Lower extremity computed tomography (CT) was considered to aid in diagnosis. CT scan with enhancement was suggestive of an oval hematoma within a thick sac (Fig. 1A and B). An elective surgery was planned.

After endotracheal general anesthesia, the patient was placed in the supine position. For accurate assessment, ultrasound scanning for mass mapping using marking pen was performed. The mass was located within the incision site on the proximal side among the two previous surgical incision sites. An incision of 7 cm on previous surgical wound revealed a huge mass (Fig. 1C). After applying self-retaining retractors to aid in exposure, we tried not to tear the shell of the mass to conserve the contained material during dissection. The mass existed exactly between the two metal clips for ligation of saphenous vein and its branches. After taking it out carefully so as not to spill its contents, we confirmed the old hematoma visually with incision on the sac (Fig. 1D). The maximum length of the mass was about 10 cm. A drainage tube was then inserted. The wound was closed in a layered fashion to reduce dead space.

Pathologic findings demonstrated chronic inflammation of fibrotic sac and hematoma inside. The patient’s postoperative course was uneventful. He was discharged on postoperative day 7 after removing the drainage tube within the surgical wound. Thirty days after the surgery, the surgical wound site was fine without recurrence at the outpatient clinic.

## Discussion

In this era, with gradual increase in the use of a no-touch technique [10, 11], the importance of managing vein harvest site complications should be emphasized [12]. Although endoscopic vein harvest has several benefits for wound complications and cosmetic aspects, there is still some debate about graft patency and clinical outcomes [13, 14]. Kim et al. [9] suggest that the use of a drainage tube in the no touch harvesting site is important in reducing complications associated with leg wounds.

There are many kinds of complications associated with vein harvesting [4, 15]. Surgical site infection is focused on the most. Many risk factors for leg wound infection have been established [2, 15]. About 70% of leg infections are diagnosed within 30 days of surgery [3]. However, to the best of our knowledge, there is no literature on huge and chronic state of hematoma with fibrotic sac at saphenous vein harvest site which required surgical intervention.

We encountered a quite rare case of hematoma within a well-demarcated sac around the previous saphenous vein harvest site. Preoperative planning was established cautiously for the huge size of the mass. The mass was adjacent to two metal clips used for saphenous ligation. As the patient fell, it is assumed that a hemorrhage occurred in a branch of the great saphenous vein. Thus, it could be presumed that foreign body reaction caused the thick fibrotic sac to occur. If the mass had been simply aspirated percutaneously, it would be more likely to recur. There are some reports of titanium inducing foreign body reactions such as inflammation and fibrosis [16, 17], but the probability is very low. So, it is not known with certainty why the large hematoma and the fibrotic sac emerged, as there is no definitive evidence to support their occurrence, only speculation.

## Conclusion

For a leg wound complication after CABG, lower extremity CT and ultrasound scanning were useful tools for preoperative assessment. Our experience suggests that a huge hematoma within thick fibrotic sac at saphenous vein harvest site can occur as a complication after CABG.

## Data Availability

As this paper is a case report, all generated or analyzed data are included in this article.
